# The Influence of Food Intake Specificity in Children with Autism on Gut Microbiota

**DOI:** 10.3390/ijms21082797

**Published:** 2020-04-17

**Authors:** Aleksandra Tomova, Katarina Soltys, Petra Kemenyova, Miloslav Karhanek, Katarina Babinska

**Affiliations:** 1 Institute of Physiology, Faculty of Medicine, Comenius University in Bratislava, 813 72 Bratislava, Slovakia; petra.kemenyova@fmed.uniba.sk (P.K.); katarina.babinska@fmed.uniba.sk (K.B.); 2 Comenius University, Science Park, Comenius University in Bratislava, 841 04 Bratislava, Slovakia; katarina.soltys@uniba.sk; 3 Department of Microbiology and Virology, Faculty of Natural Sciences, Comenius University in Bratislava, 842 15 Bratislava, Slovakia; 4 Department of Molecular Biology, Faculty of Natural Sciences, Comenius University in Bratislava, 842 15 Bratislava, Slovakia; 5 Biomedical Research Center, Slovak Academy of Sciences, 845 05 Bratislava, Slovakia; miloslav.karhanek@savba.sk

**Keywords:** autism spectrum disorder, intestinal microbiota, food intake

## Abstract

Autism spectrum disorder (ASD) is a complex of neurodevelopmental conditions with increasing incidence. The microbiota of children with ASD is distinct from neurotypical children, their food habits are also different, and it is known that nutrient intake influences microbiota in a specific way. Thus, this study investigates the food habits of children with ASD and their association with the gut microbiota. Children with ASD had their dietary energy intakes similar to controls, but they more often demonstrated food selectivity, which seemed to result in deficiency of micronutrients such as vitamins K, B6, C, iron, cooper, docosahexaenoic and docosapentanoic acid. Using high-throughput sequencing, a DNA library of intestinal microbiota was performed. Core microbiota was similar in children with and without ASD, but *Dichelobacter*, *Nitriliruptor* and *Constrictibacter* were found to be putative markers of ASD. The changes in gut microbiota that we observed in connection to food selectivity, intake of fats and omega-3 in particular, fermented milk products and animal/plant protein consumption had similar character, independent of diagnosis. However, high fibre intake was connected with a decreased α-diversity only in children with ASD. High carbohydrate and fibre intake influenced β-diversity, changing the abundance of *Bacteroides* and other genera, many of them members of the *Clostidiaceae*. Modulating food habits of ASD children can influence their gut microbiota composition.

## 1. Introduction

Autism spectrum disorder (ASD) is a complex of neurodevelopmental conditions with increasing prevalence. A large proportion of children with ASD are reported to have food selectivity. They refuse to eat a diet based on a variety of foods and consume a narrower food repertoire consisting of about two-thirds the number of foods as typically developing children [1,2]. Sensory selectivity, based on tactile/texture, gustatory, and olfactory oversensitivity, is proposed to contribute to the development of food selectivity [1]. Additionally, children with ASD are often subjected to some type of dietary intervention and elimination of some foods or food groups [3]. These feeding habits raise the question of nutritional adequacy [1]. As an example, it was shown that children with ASD consume fewer proteins and more carbohydrates than the general population [4], and their diets may be low in micronutrients [4,5]. The most frequently omitted food group is vegetables, followed by fruits [6]. Evidence shows that feeding problems are associated with gastrointestinal (GI) and intestinal microbiota change in children with ASD. It has been established for the general population and confirmed using animal models that the consumption of particular diets shifts gut microbiota to specific bacterial genera [7]. In particular, a high-fat diet deceases α-diversity, *Firmicutes/Bacteroidetes* ratio, *Blautia* and *Faecalibacterium* abundance and increases *Alistripes* and *Bacteroides* abundance [8]. Supplementation of omega-3 fatty acids has been shown to temporarily shift intestinal microbiota towards bacterial species producing short-chain fatty acids [9]. Dietary intake of non-fermentable fibre is reported to increase such genera as *Helicobacter*, *Enterococcus*, *Desulfovibrio*, *Parabacteroides*, *Pseudoflavonifractor* and *Oscillibacter*, while decreasing genera such as *Lactobacillus*, *Parasutterella*, *Coprobacillus* and *TM7 genera Incertae Sedis*. This leads to changes in the metabolic profile that are beneficial for the prevention of autoimmune diseases [10]. Recent meta-analysis including studies with different kinds of dietary fibre concluded that fibre intake leads to a higher faecal abundance of *Bifidobacterium* and *Lactobacillus* spp. but does not affect α-diversity [11].

The amount of a specific macronutrient in the diet, as well as its source, is important for the microbial configuration of host intestines. Type, composition and quantity of dietary proteins are associated with specific intestinal microbiota, thus they influence microbial metabolites in the intestine, and modulate the function of intestinal barrier and host immune defence [12]. High animal protein intake increases the *Bacteroides/Prevotella* ratio and even changes the microbial enterotype of the host. Furthermore, the nature of food intake affects intestinal microbiota. but in response, the intestinal microbiota itself can regulate eating habits, stimulating the intake of foods beneficial to them and suppressing competitors [13]. Thus, the feeding habits of children with ASD raise several questions including the role of nutritional inadequacy of the diet, the connection of food composition with GI disorders, and the consequences in terms of a change in their intestinal microbiota.

In addition to the above mentioned, it is known that gut microbiota in children with ASD is different compared with control children [14,15,16]. The ratio of the main phyla *Bacterodetes*/*Firmicutes* switches, and genera such as *Clostridium, Sutterella, Lactobacillus*, *Nitriliruptor, Youngiibacter, Methanomicrobiales, Bilophila* and *Desulfovibrio* change in abundance. This change displays an association with behavioural manifestations. This association suggests bidirectional influence of microbiota and brain, explaining the recent interest in the microbiota–gut–brain axis [17]. The different pathways involve the leakage of bacterial metabolites into the blood, and these, through cascades of reactions, alter the tight junctions in the blood–brain barrier, cortex, hippocampus, amygdala and cerebellum [18]. A recent review of nutritional interventions for gut microbiota modulation in ASD [19] has revealed that in this area, the gaps in knowledge are greater than what is known.

Taken together, the specific microbiota reacts to an individual’s diet in a personalised way [20]. The interplay between diet, microbiota and host, in children with ASD along with their distinct microbiota, would probably be different from that of neurotypical children.

Thus, this aim of our study to make a step towards elucidating the connection of eating disorders and specificity of food intake, with intestinal microbiota composition in children with ASD, keeping in mind that some microbiota changes might be associated specifically with their neurodevelopmental disorder.

## 2. Results and Discussion

Participants in the children with ASD group were chosen based on a confirmed diagnosis as described in the Methods section. Children in the control group had similar rates of GI complaints, suggesting that possible differences in microbiota are not associated with GI symptoms. The parameters and criteria used to compare microbiota, such as food selectivity, high-fibre diet etc., within the group of children with ASD were chosen based on the current literature of nutritional and feeding habits’ impact on gut microbiota.

### 2.1. Comparison of Feeding Habits and Intestinal Microbiota of Children with and without ASD

Mealtime behaviours of the individuals are presented in Table 1. Children with ASD displayed mealtime problems more frequently than controls (76.1% vs. 43.8%, *p* = 0.017). Although children with ASD have been reported to have more GI complaints compared to neurotypical children [21], in this study no significant differences either in frequency or in severity of GI disorders were observed between the ASD and control group (Table 1.). Of the most commonly presented GI symptoms, which include bloating, abdominal pain, and constipation, significant differences were observed only in the prevalence of constipation in ASD (28.3% vs. 0%, *p* = 0.014).

Feeding problems are also common in children with ASD [22]. In our study, the group of children with ASD contained significantly more individuals who displayed food selectivity (“picky eaters”) compared to controls (57.7% vs. 25%, *p* = 0.02). Children presenting with selectivity prefer limited food choice, stereotypes during meals, and also significantly more often demonstrate aggressive behaviour or other behavioural disorders during the food intake. Aggressive behaviour, that may also adversely affect their food intake and the variety of food consumed, was observed in more than one third of children with ASD, but in none of the controls (*p* = 0.006). However, energy intake did not differ between the groups, and correspondingly, no significant differences in age-specific values of BMI (SDS BMI) were observed between controls and children with ASD (Table 1). The majority of the study participants fell in the normal range for BMI, as earlier reported [6]. Obesity (SDS BMI > 2 SD) was observed only in one child with ASD, and two control children.

Upon analysing specific nutrient intake, no significant differences in macronutrients were found between the studied groups (Table 2). Similarly, intakes of essential and non-essential amino acids, saturated and unsaturated fatty acids, and total sugars (mono and disaccharides) did not differ between the groups. However, children with ASD had significantly lower intake of docosahexaenoic acid, docosapentanoic acid, iron, cooper, iodine, and vitamins K, B6, and C (data not presented). This suggests a risk of inadequate nutrient intake compared to neurotypical children. Sharp et al. in 2018 identified a decreased daily intake of several nutrients in subjects with ASD when compared to the recommendations [6]. Notably, the abovementioned study found different nutrients to be consumed in lower amounts compared to our study, except for iron, which was lower in both studies.

**Figure 1:**
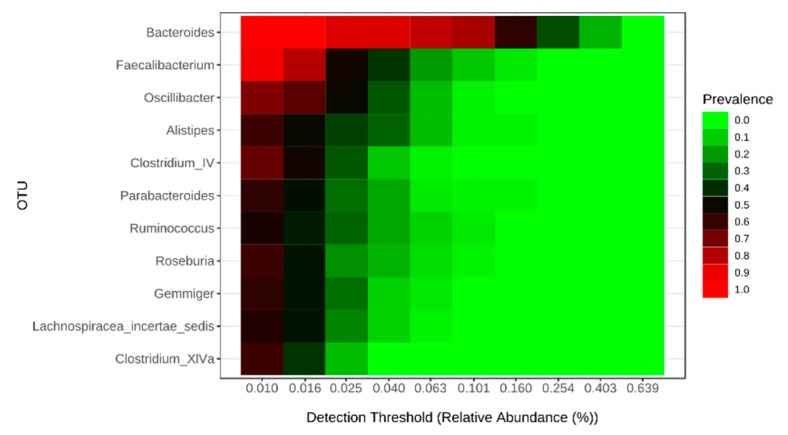
Core intestinal microbiome in children with and without ASD.

**Figure 2:**
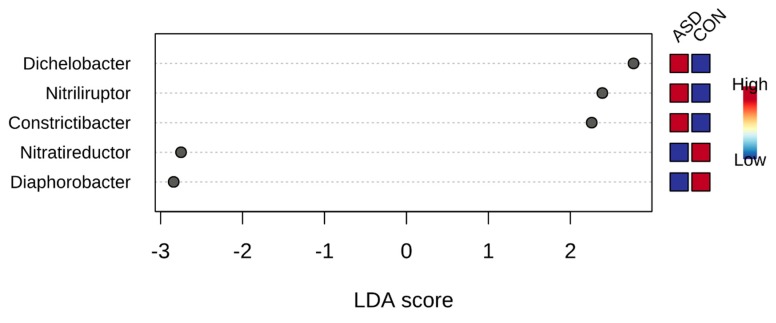
Microbiota biomarkers for ASD children compared to neurotypical, calculated with linear discriminant analysis effect size (LEfSe), MicrobiomeAnalyst.

Before investigating specific food intake influence on gut microbiota in children with ASD, we compared the gut microbiota of children with and without this disorder. Notably, despite the difference in nutrition, both groups had the same core microbiota (Figure 1), and alpha diversity was not different in children with and without ASD. On the other hand, using linear discriminant analysis effect size(LEfSe), we found univocal biomarkers in the gut microbiota, particularly *Dichelobacter*, *Nitriliruptor* and *Constrictibacter* were typical for ASD group, while *Diaphorobacter* and *Nitratireductor* were typical for the control group (Figure 2). Thus, these bacterial genera were assumed to characterize the differences between children with autism and neurotypical children, rather than to characterize the specificity of the feeding habits.

### 2.2. Food Selectivity Reflects the Faecal Microbiota Composition

Previous studies showed that children with ASD included significantly more “picky eaters” when compared with controls. The prevalence of food selectivity in our sample of children with ASD corresponded to observations of other authors [2]). Food selectivity correlated with behavioural problems during feeding in ASD children (Pearson correlation 0.64, *p* < 0.0001). Children with autism also had behavioural disorders during feeding more frequently than controls (7.17 vs. 2.68, *p* = 0.0006). Among the children with ASD, “picky eaters” had more GI disorders compared to “non-picky eaters” (total GI score 6.63 vs. 3.6, *p* = 0.02) with belly pain (average score 0.73 vs. 0.25, *p* = 0.035) and constipation (average score 1.19 vs. 0.05, *p* = 0.001) significantly more often. Within the group of children with ASD, “picky eaters” displayed a correlation between GI score and the social interaction scale (A-SUM) of ADI-R (Autism Diagnostic Interview-Revised) (*p* = 0.02, Pearson R = 0.46), which was not seen in non-picky eaters. Thus, these data suggest that "picky eating" is associated with GI and behavioural disorders in children with ASD.

**Figure 3:**
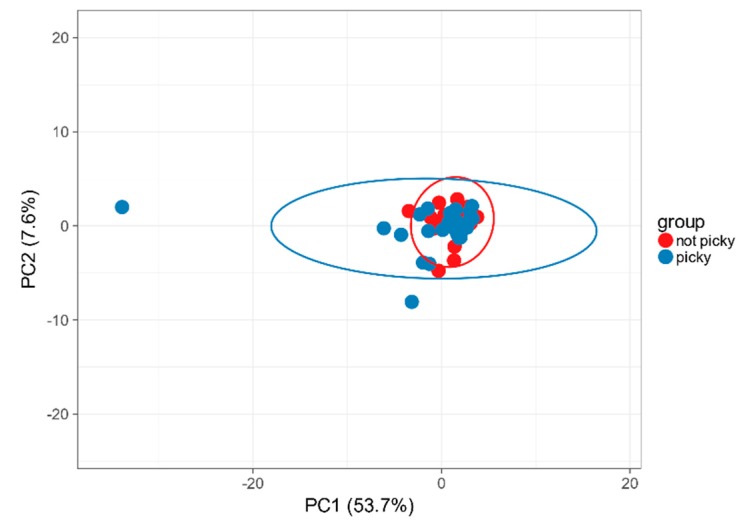
Diversity of intestinal microbiota at the phylum level in “picky eaters” compared to “non-picky eaters”. ClustVis PCA.

Analysis of the intestinal microbiota at the phylum level in the ASD group displayed more heterogenous composition in “picky eaters” compared to “not picky eaters” (Figure 3). Six out of 58 phyla were significantly differently abundant and another seven tended to be differently abundant in children with and without food selectivity. The ratio of the most represented phyla *Bacteroidetes*/*Firmicutes* was higher in “picky eaters” compared to “non-picky eaters” (3.34 vs. 1.78), though it was only a trend, based mainly on *Bacteroidetes* abundance.

**Figure 4:**
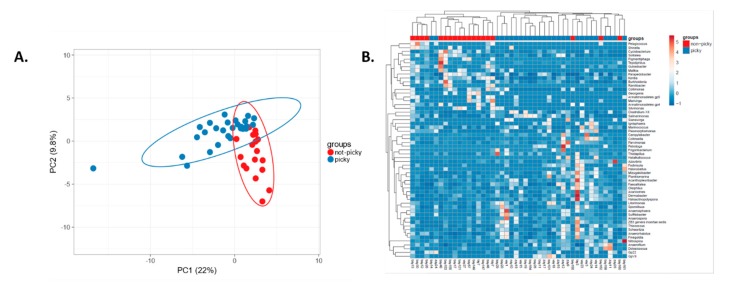
Diversity of intestinal microbiota at the genus level in “picky eaters” compared to “non-picky eaters”. **A**. PCA ClustVis. **B**. Heatmap ClustVis, significantly differently abundant genera.

We found that 58 genera were significantly differently abundant in children with ASD from different subgroups based on the presence or absence of food selectivity. Food selectivity makes faecal microbiota of “picky eaters” distinguishable from “non-picky eaters” within children with ASD (Figure 4). Representatives of *Enterobacteriacaea*, *Escherichia*/*Shigella* and *Salmonella*, from *Proteobacteria*, as well as *Clostridium* XlVa, *Anaerofilum* from *Clostridia*, *Firmicutes*, were characteristic of “picky eaters”, according to LEfSe. These genera could also be associated with GI discomfort. Importantly, the most typical genera for ASD "picky eaters" were the same as for neurotypical "picky eaters" (data not shown). *Prevotella*, *Bacteroides*, *Parabacteroides* and *Bacteroidetes* characterised “non-picky eaters”. *Bacteroides* are often associated with elevated meat intake, while studies connect *Prevotella* with plant-based diets. Both have been described as having pro-inflammatory effects [23]. In our participants, the core microbiota did not depend on food selectivity, dietary fibre, vegetable, fermented milk intake or any other studied criteria.

### 2.3. Carbohydrate Intake and Intestinal Microbiota

**Figure 5:**
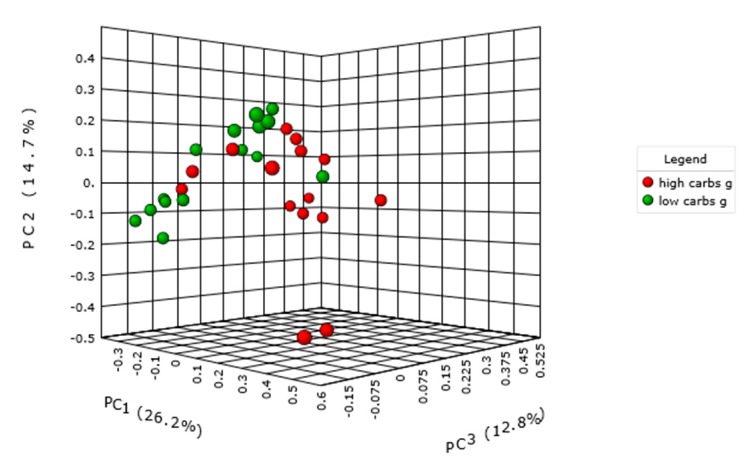
Intestinal microbiota diversity in children with ASD with high and low carbohydrates intake per day, *p* = 0.01.

Consumption of more than 180 g/day was considered a high carbohydrate intake, and subjects with lower consumption were classified as having low carbohydrate intake. We did not observe an impact on the microbiome alpha diversity of individuals. However, within the cohort of children with ASD, we found a significant difference in microbiota relative abundance between the high and low carbohydrate intake subgroups (*p* = 0.01) (Bray-Curtis index, Permanova) (Figure 5). This was not different in samples of neurotypical children. One of 65 genera that significantly differed in their abundance were *Bacteroides*; their copy number was 2.5 times lower in children with higher intake of carbohydrates, as was previously observed in the neurotypical population. The most abundant genera that significantly differed along with *Bacteroides* included *Oscillibacter, Flavonifractor, Intestinimonas* and *Pseudoflavonifractor* as well. *Lactococcus* was increased with higher carbohydrate intake. Since the difference was found only in the ASD group, we investigated the intake of the dietary fibre as a specific carbohydrate, as well as the consumption of vegetables and fruit.

Higher carbohydrate intake in children with ASD significantly increased the score in social affect, and it displayed a trend with an increased score in reciprocal social interaction and total raw score of ADOS-2 (Autism Diagnostic Observation Schedule, Second Edition).

### 2.4. Dietary Fibre Intake and Intestinal Microbiota

**Figure 6:**
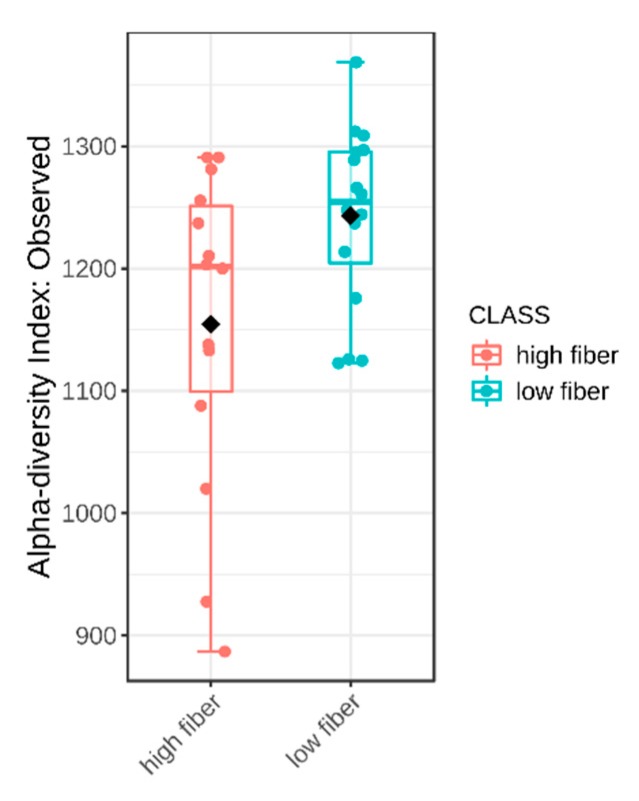
Alpha diversity of intestinal microbiota in children with ASD with high and low daily fibre intake, *p* = 0.037.

**Figure 7:**
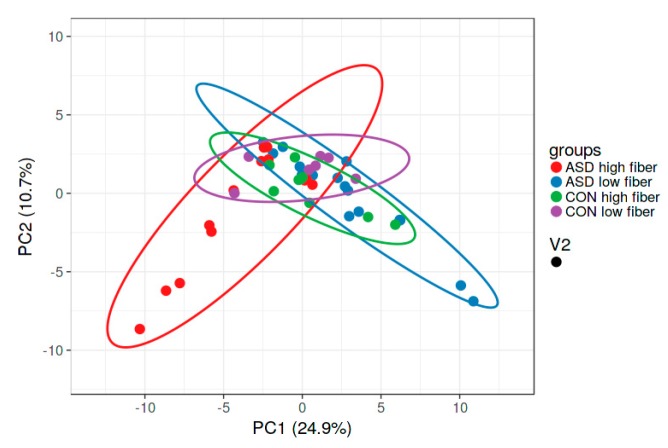
Visualisation of intestinal microbiota diversity at genera level of neurotypical children and children with ASD with high or low daily fibre intake (ClustVis).

**Figure 8:**
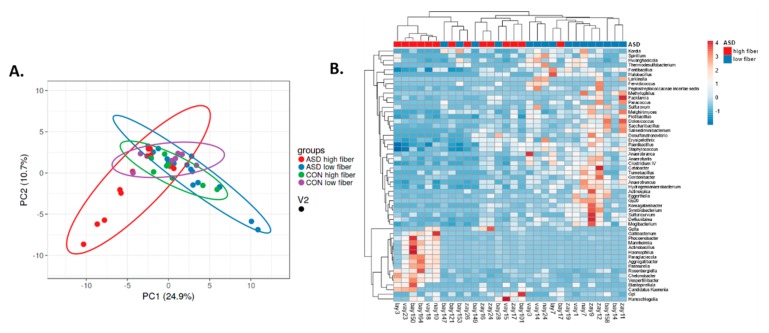
Intestinal microbiota diversity in children with ASD with high or low daily fibre intake, ClustVis. **A**. PCA. **B**. Heatmap, significantly differently abundant genera.

Dietary fibre comprises edible carbohydrate polymers with three or more monomeric units. It is resistant to endogenous digestive enzymes in the gut and many studies have proven its influence on the intestinal microbiota [24]. In our study, the fibre intake did not differ significantly between the children with and without autism. However, within the ASD group, alpha diversity was significantly lower in the subgroup of children with high fibre intake (Figure 6), unlike the controls, where there was no difference. This observation was not expected, since it opposes the irreversible reduction in microbial diversity in low dietary fibre diet described earlier [25,26]. Nevertheless, a meta-analysis found no change in α-diversity in increased fibre intakes [11]. Children with ASD have increased microbial diversity in the intestine when compared with controls [14], and this can be crucial for the change in microbial diversity under the influence of nutrient intake. Our study observed higher β- diversity in both ASD subgroups compared with both control subgroups. In the ASD group, microbiota in children with higher fibre intake per day notably differed from this in children with lower fibre intake (Figure 7). Seventy-three genera were significantly differently abundant (Figure 8). Low fibre intake significantly increased the abundance of *Hydrogenoanaerobacterium*, *Clostridium* IV, *Anaerotruncus* from *Clostridiaceae* and others. Increased fibre intake decreased the GI score in children with ASD, i.e., it was associated with lower frequency of GI disorders (data not shown), which suggests fibre’s importance for healthy GI functioning.

Although the daily fibre intake did not reach a significant difference between children with ASD and neurotypical children, fresh fruit and vegetable intake was significantly higher in neurotypical children compared to children with ASD, and this was expected [6]. Dietary fibres derived from fruits and vegetables, compared with that from cereal, contain a considerably higher proportion of soluble fibres, which exhibit better fermentability, and they have been shown to increase microbiota diversity and change the gut microbiota composition [24]. In our study, α-diversity was higher with higher intake of fresh vegetables than in the control group, but not in the group of children with ASD (data not presented). This, however, may be due to the generally low intake of vegetables in children with ASD. As expected, a high intake of vegetables significantly increased *Bacteroides* and *Hungatella* abundance.

According to the recommendations [27], adequate fibre intake for children is 14 g/1000 kcal (4184 kJ) of energy consumed. In our samples, the average intakes were just above half of the recommended quantity both in children with ASD (8.00 ± 3.26 g, mean, standard deviation) and in the control group (8.57 ± 1.67 g).

This amount of fibre may lead to the disappearance of specific bacterial species and this reduction is insufficient for improving the inflammatory status [25], a status often elevated in children with ASD [28]. Moreover, non-fermentable fibre helps to tune the immune status by the intestinal microbiota and so prevents autoimmune neurological disease [10]. Thus, it is recommended to increase fibre intake in both investigated groups, but especially in children with ASD.

### 2.5. Fermented Milk Intake and Intestinal Microbiota

**Figure 9:**
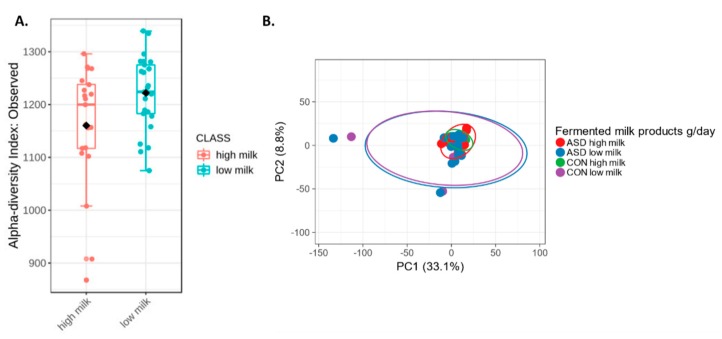
Alpha-diversity (**A**) and beta-diversity (**B**) of intestinal microbiota in children with high and low daily intake of fermented milk products.

An interesting finding of our study is the effect of consumption of fermented milk products on intestinal microbiota. Alpha diversity was significantly higher in children consuming lower quantities of fermented milk products, independently of diagnosis (Figure 9A). Beta-diversity showed differences in microbiota between fermented milk subgroups (higher intake vs. lower intake) of children from both ASD and the control group (Figure 9B). Subgroups of ASD children with low milk intake had significantly increased abundance of *Butyricimonas, Anaerotruncus, Guggenheimella, Acetanaerobacterium, Vallitalea* and other bacteria, most of which belong to class *Clostridia*. In our study, *Lactobacillus, Blautia, Anaerostipes* and *Fusicatenibacter* were typical for children with autism who consumed more fermented milk products (LEfSe), as expected from earlier studies [29,30]. *Fusicatenibacter*, like *Lactobacillus,* produces lactic acid, acetic acid and succinic acid [31]. The control group, unlike ASD, has *Sporomusa* and *Haemophilus.* Our results suggest that fermented milk consumption changes microbial community structure in the gastrointestinal tract, as suggested before [32], but these alterations depend on the type of bacteria in fermented milk [33] and seems to depend on the background intestinal microbiota.

### 2.6. Omega-3 Intake and Intestinal Microbiota

**Figure 10:**
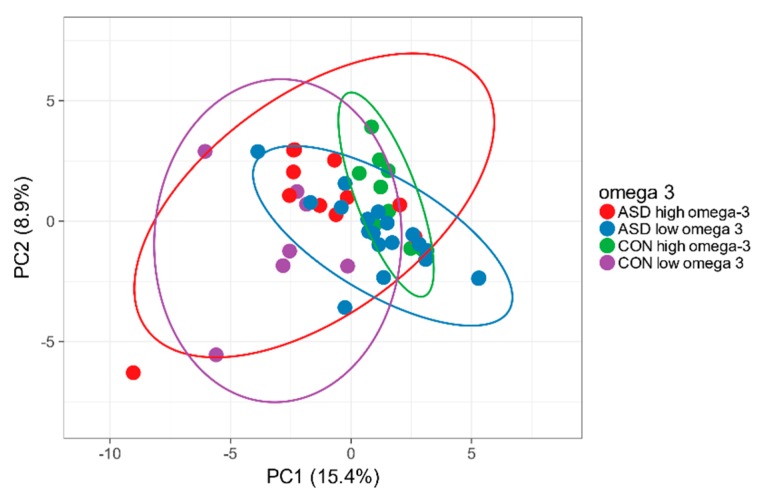
Intestinal microbiota in children with and without ASD with high and low omega-3 daily intake, significantly differently abundant genera.

No difference in omega-3 intake in children with and without ASD was found. There were no significant differences in α and β diversity in higher and lower total fat or omega-3 intake in particular in ASD or in control children, as we expected [8]. Children with ASD who had higher omega-3 intakes had significantly higher abundance of *Catonella, Coprobacter, Marvinbryantia* genera, found normally in the faeces of healthy people. We did not observe changes in *Bifidobacterium*, *Roseburia*, *Lactobacillus* or others that were identified in previous studies [9,34]. The reason for this could be a natural source for omega-3 fatty acids from the food, reaching the maximum of 1.52g/day, while in the mentioned article, the subjects were supplemented with 4g omega-3 PUFA (polyunsaturated fatty acids)s per day. The microbiota profile in high and low omega-3 intake subgroups of children with autism and controls is shown in the Figure 10.

Docosahexaenoic acid intake was different in children with ASD and controls, but high intake was not associated with significant differences in gut microbiota compared to low intake.

### 2.7. Animal vs. Plant Protein Intake

Preferences for plant protein were significantly associated with decreased GI complaints (*p* = 0.02). No differences in diversity were found between the investigated subgroups. *Flavonifractor* from *Clostridia* was the most abundant taxa and significantly increased in children with ASD, who preferred animal proteins. This is rarely isolated from clinical human specimens, and the literature shows an association with the risk of cholecystitis [35] and colorectal cancer [36]. The expected change in the ratio of *Prevotella* to *Bacteroides* [25] was not observed.

The complexity in the interpretation of gut microbiota analysis as well as the difficulties of possible intervention in response to it are based on unique diets, reflected by the unique microbiota of each individual. The limitations facing all investigations of intestinal microbiota, including study, is its dependence on many factors, as age, geography, diet etc. Fortunately, it has been shown that habitual dietary patterns stabilize faecal microbiota in children from 4 to 8 years [37], which makes the findings of this investigation reliable. Limitations of this study imclude the low number of participants and the subjectivity of food intake tests.

Nevertheless, additional studies are needed to confirm the effects discovered in our study.

## 3. Materials and Methods

The study included 62 boys, of which 46 had ASD and 16 were control non-autistic children. The characteristics of the groups are included in Table 1. Written informed consent was obtained from parents of all participating children.

Children with ASD were recruited from the Academic Research Centre for ASD (ARCA) based at the Institute of Physiology, Faculty of Medicine Comenius University in Bratislava, Slovakia. Psychological evaluation of children with ASD was performed using the ADOS-2 (Autism Diagnostic Observation Schedule, Second Edition) [38] and ADI-R (Autism Diagnostic Interview, Revised) [39] behavioural assessment scales, which are internationally accepted gold standards for the diagnosis of ASD. ADOS-2 evaluated the behaviour in domains of social affect (SA), distinguishing communication (COM) and reciprocal social interaction (RSI), restricted and repetitive behaviour (RRB) scores, and total raw score (Total). ADI-R was evaluated in the areas of qualitative abnormalities in reciprocal social interaction (A) and communication (B), as well as in restricted, repetitive and stereotyped patterns (C) of behaviour. All subjects involved in the study met the criteria for ASD using both diagnostic tools. The diagnosis of ASD met the criteria for DSM-V (The Diagnostic and Statistical Manual of Mental Disorders, Fifth Edition). Control subjects were recruited from local kindergartens and schools and had no psychiatric conditions according to their parent interview. Written informed consent was obtained from parents of all participating children. The protocol was approved by the Ethics Committee of the Comenius University Faculty of Medicine and the University Hospital, APVV 15-0085 approved on 13.06.2016. The study conformed to the code of ethics stated in the Declaration of Helsinki.

Data on gastrointestinal (GI) status and mealtime behaviours were evaluated based on the parental questionnaires. The prevalence and frequency of gastrointestinal symptoms (abdominal pain, bloating, constipation, diarrhoea, hard stools, pain during defecation, voluminous stools) were obtained. Based on their prevalence, the GI score was calculated; the higher the value, the more prevalent the GI problems were. BMI was calculated as body weight in kilograms/square root of height in meters. BMI standard deviation scores (BMI-SDS) were calculated using the reference data of the Slovak population in order to determine the deviation in BMI from the mean BMI of the general population of children of the same age and gender [40]. The prevalence and frequency of adverse mealtime behaviours was recorded, including selectivity in food intake, as well as anger, crying or self-injuries associated with food intake. Child insistence on having the food prepared and served in the same manner was defined as stereotyped behaviour during mealtimes. The score of mealtime problems was calculated based on their prevalence. Higher values indicated more severe mealtime problems. Data on modifications of the child’s diet were recorded, as well as information about the use of food supplements in the last 12 months.

Of the total 62 individuals, data from 30 children with ASD and 16 controls were collected about the typical diet of the subjects by a self-administered food frequency questionnaire (FFQ) validated for the Slovak population that included 85 food items. Parents of children were requested to indicate the frequency of consumption of each food item (with options including times per day, times per week, times per month, or almost never) together with the portion size. Average daily consumption of selected food types (g/day) was calculated. Food intake data served for the calculation of the nutrient intake of the individuals, vegetable and fruit intake was adjusted for seasonality. For the conversion of food intake into nutrient intake, the Slovak food composition database was used (Slovak Food Composition Bank. Revision 2004 (2004). Food Research Institute, Bratislava 2004) (http://www.vup.sk/en/index.php?navID=25?start). Calculations were performed using Microsoft Excel standard formulas. 

Stool samples were collected by parents at home in sterile flasks. Parents were given a detailed explanation of the procedure and stored the samples at + 4 °C after collection. Samples were delivered to the laboratory within 4 hours and divided into 2 aliquots, one of which was immediately frozen at −80 °C for future DNA purification of intestinal microbiota assessment and the other frozen at −20 °C for calprotectin investigation.

DNA was extracted from frozen stool samples using a commercial extraction system (QIAamp DNA Fast Stool Mini Kit, Qiagen, Hilden, Germany) according to the manufacturer’s instructions. The DNA concentration was determined using the NanoDrop 2000 Spectrophotometer (ThermoFisher Scientific, MA, USA).

High-throughput sequencing was performed for the DNA libraries. The PCR amplification of V1-V9 region of 16S rRNA using primer set 27f / 1492r was carried out using the 27f (5’-AGA GTT TGA TCM TGG CTC AG-3’) and the 1492r (5’-CGG TTA CCT TGT TAC GAC TT-3’) primers (Lane, 1991). In this process, 3-50 ng of total input DNA in 20 µL volume PCR reaction was amplified with 4µl of 5x HOT FIREPol Blend Master Mix (Solis BioDyne, Tartu, Estonia), 0.4 µL (10 µM) of each primer (final concentration 0.2 µM) and milli-pore water. PCR conditions were as follows: initial denaturation 95 °C/15 min, cycling 25× (95 °C /20 sec, 60 °C/30 sec, 72 °C/2 min), final polymerization 72 °C/10 min. Amplicons were column-purified (Zymo DNA Clean and Concentrator-5, Zymo Research, Irvine, CA, USA) according to standard protocols and quantified fluorometrically with Qubit™ dsDNA HS Assay Kit (Thermo Fisher Scientific, Waltham, MA, USA). Amplicon sequences were fragmented by a transposon-based approach (Nextera XT, Illumina San Diego, CA, USA) and low-cycle PCR and mutual indexing of the fragments was performed. Fragment size selection and purification with 1.8x AMPure XP beads yielded final DNA libraries that were verified using the Agilent 2100 Bioanalyzer (Agilent Technologies, Waldbronn, Germany) and quantified using the Qubit 2.0 Fluorometer (Thermo Fisher Scientific, Waltham, MA, USA). The 4 nM pool of libraries was further diluted to 10pM and sequenced on Illumina MiSeq platform (Illumina, San Diego, CA, USA) with 2 × 300 bp paired-end sequencing at the Comenius University Science Park (Bratislava, Slovakia). Library sequence data were quality checked using FastQC, Andrews, 2010, a quality control tool for high throughput sequence data available online at: http://www.bioinformatics.babraham.ac.uk/projects/fastqc. Further data processing, including trimming, 16S analysis and visualization, was performed with Geneious (Biomatters Ltd, Auckland, New Zealand). For visualizing and clustering of multivariate data using principal component analysis (PCA) ClustVis (https://biit.cs.ut.ee/clustvis/) [41] and MicrobiomeAnalystR [42,43] (https://github.com/xia-lab/MicrobiomeAnalystR) were applied. For diversity calculations, the Bray-Curtis Index and Permanova test were used.

Data were presented as mean ± SEM values and p values lower than 0.05 were accepted as significant. Since the data distribution matched Gaussian distribution for the correlation analysis, the Pearson correlation coefficient was applied. For all statistical analyses, GraphPad Prism 5 and Microsoft Excel 2016 were used. Data on food intake did not pass the normality test, therefore they are presented as median ±95% CI (confidence interval), and their differences were tested by the Mann–Whitney test. For the testing of categorical variables, the chi-square test and Fisher’s exact test were used.

High carbohydrate, protein and fat intake corresponded to the consumption of more than 180, 40 and 50 g/day, respectively. High fibre, fresh vegetables and fresh fruit intake corresponded to the consumption of more than more than 10, 40 and 150 g/day, respectively. High fermented milk or white bakery intake corresponded to the consumption of more than 100 or 40 g/day, respectively. High omega-3 (linolenic, eicosapentaenoic, docosahexaenoic acids) intake corresponded to the consumption of more than 0.75 g/day.

## 4. Conclusions

Interest in the modulation of intestinal microbiota by specific food intake has increased in recent years. The degree to which eating habits influence gut microbiota in children with ASD is not clear as of yet. Children with ASD frequently have eating and GI disorders, which influence the intestinal microbiota and clinical manifestations and vice versa, with the microbiota influencing eating habits, GI status and behavioural disorders. Our study shows that nutritional inadequacies in children with ASD could be hidden behind normal BMI. Food selectivity, found more frequently in children with ASD, resulted in different micronutrient intake as compared with controls. Although the core microbiota composition was independent of diagnosis or specific nutrient intake, ASD group microbiota was characterised by *Dichelobacter, Nitriliruptor* and *Constrictibacter*, while *Diaphorobacter* and *Nitratireductor* were typical for the control group. For “picky eaters”, the gut microbiota was more diverse at the phylum level, distinct at the genera level and characterized by specific genera (*Escherichia/Shigella* and *Salmonella, Clostridium XlVa*, *Anaerofilum*). Changes in gut microbiota were similar in children with and without ASD. Higher carbohydrate intake changed beta diversity only in children with ASD, for example by decreasing *Bacteroides* abundance. Fibre intake was similar in groups, but increasing it in children with ASD decreased microbial diversity. Fresh fruit and vegetable intake were significantly higher in neurotypical children compared to children with ASD, and their consumption increased microbiota diversity exclusively in the control group. High intake of fermented milk products had strong effects, that were similar in both groups: they increased *Lactobacillus* and *Fusicatenibacter* abundance and decreased microbial diversity. Distinct microbiota were found when omega-3 consumption with food was increased in both groups. Aside from bacteria characterising the microbiota in autism, the reflection of microbiota to nutrients intake was similar in both groups, with the exception of fibre intake.

Food selectivity, as well as the consumption of fermented milk products, total fat, omega-3, animal/plant protein resulted in similar changes in the intestinal microbiota of children with and without autism. However, the effects of carbohydrates, fibre, fruits, vegetables intake were different.

Thus, although food intervention in children with ASD is difficult, such changes could help to alter the intestinal microbiota in such a way as to improve GI and immune status.

## Figures and Tables

**Table 1:** Gastrointestinal symptoms and nutrition-related characteristics of cohorts of individuals with autism spectrum disorder (ASD) and neurotypical controls.

	ASD	Controls	p
N (all boys)	46	16	
Age range (years)	4.0–8.5	2.8–9.15	
Age (mean ± SD)	6.3 ± 1,5	5.1 ± 1.7	0.017
BMI (kg.m^-2^)	17.1 ± 3.7	16.2 ± 2.0	0.746
SDS BMI	0.12 ± 0.98	0.50 ± 1.56	0.781
Mealtime problems (% of subjects) of that	76.1%	46.8%	0.017
Food selectivity	58.7%	25%	0.020
Aggressive behaviour	34.8%	0.0%	0.006
Stereotyped behaviour	32.6%	18.8%	0.168
Presence of GI symptoms (% of subjects) of that	89.4%	87.5%	0.838
Constipation	28.9%	0.0%	0.014
Diarrhoea	2.2%	6.3%	0.437
Bloating	35.6%	56.3%	0.148
Abdominal pain	35.6%	25.0%	0.439
Pain upon defecation	17.8%	6.3%	0.284
Dietary restrictions initiated by parents	17.4%	0.0%	0.099
Food supplement intake	76.1%	93.8%	0.123

**Table 2:** Daily intakes of energy, selected nutrients and foods in cohorts of individuals with ASD and neurotypical controls.

	ASD	Controls	p
N (all boys)	30	16	
Energy and nutrients (mean ± SD)			
Energy, kJ/day	5506 ± 254	5666 ± 344	0.711
Proteins, g/day	45.5 ± 0.4	50.2 ± 0.9	0.273
Animal/plant protein	1.63 ± 0.03	1.76 ± 0.04	0.557
Fats, g/day	53.1 ± 0.6	55.5 ± 1.0	0.656
Carbohydrates, g/day	173.1 ± 1.3	173.9 ± 2.7	0.952
% energy from protein	14.2 ± 0.3	15.0 ± 0.47	0.148
% energy from fat	35.7 ± 1.1	36.8 ± 1.1	0.536
% energy from carbohydrates	50.1 ± 1.2	48.1 ± 1.3	0.306
Dietary fibre, g/ day	9.95 ± 0.56	11.7 ± 1.00	0.114
Omega-3 fatty acids (g/day) of that	0.75 ± 0.01	0.81 ± 0.01	0.473
Linolenic acid (g/day)	0.73 ± 0.01	0.78 ± 0.01	0.548
Eicosapentaenoic acid (mg/day)	10.0 ± 0.60	20.0 ± 1.00	0.325
Docosahexaenoic acid (mg/day)	7.0 ± 0.20	13.0 ± 0.40	0.022
Foods (median, 95% CI)			
Bakery products white (g/day)	62.5 (38.7–90.0)	60.5 (21.4-65.4)	0.037
Bakery products wholegrain (g/day)	4.8 (0.0–21.4)	21.4 (7.1-53.6)	0.102
Fermented milk products (g/day)	125.9 (19.3–244.2)	67.5 (42.9–109.3)	0.393
Fresh fruit (g/day)	84.1 (47.8–133.9)	199.1 (126.0–237.3)	0.001
Fresh vegetables (g/day)	16.6 (2.9–26.8)	61.4 (36.2–95.1)	0.000
